# Identification of *TP53BP2* as a Novel Candidate Gene for Primary Open Angle Glaucoma by Whole Exome Sequencing in a Large Multiplex Family

**DOI:** 10.1007/s12035-017-0403-z

**Published:** 2017-02-01

**Authors:** Shazia Micheal, Nicole T.M. Saksens, Barend F. Hogewind, Muhammad Imran Khan, Carel B. Hoyng, Anneke I. den Hollander

**Affiliations:** 1 Department of Ophthalmology, Donders Institute for Brain, Cognition and Behaviour, Radboud University Medical Center, Nijmegen, the Netherlands; 2 Department of Clinical Genetics, Academic Medical Centre, Amsterdam, the Netherlands; 3 Department of Human Genetics, Donders Institute for Brain, Cognition and Behaviour, Radboud University Medical Center, Nijmegen, the Netherlands

**Keywords:** Primary open angle glaucoma, Whole exome sequencing, *TP53BP2*

## Abstract

Primary open angle glaucoma (POAG) is a major type of glaucoma characterized by progressive loss of retinal ganglion cells with associated visual field loss without an identifiable secondary cause. Genetic factors are considered to be major contributors to the pathogenesis of glaucoma. The aim of the study was to identify the causative gene in a large family with POAG by applying whole exome sequencing (WES). WES was performed on the DNA of four affected family members. Rare pathogenic variants shared among the affected individuals were filtered. Polymerase chain reaction and Sanger sequencing were used to analyze variants segregating with the disease in additional family members. WES analysis identified a variant in *TP53BP2* (c.109G>A; p.Val37Met) that segregated heterozygously with the disease. In silico analysis of the substitution predicted it to be pathogenic. The variant was absent in public databases and in 180 population-matched controls. A novel genetic variant in the *TP53BP2* gene was identified in a family with POAG. Interestingly, it has previously been demonstrated that the gene regulates apoptosis in retinal ganglion cells. This supports that the *TP53BP2* variant may represent the cause of POAG in this family. Additional screening of the gene in patients with POAG from different populations is required to confirm its involvement in the disease.

## Introduction

Glaucoma is a leading cause of irreversible blindness worldwide, affecting more than 60 million people around the world [1]. Glaucoma comprises a group of heterogeneous optic neuropathies, characterized by progressive optic nerve degeneration. The diagnosis of glaucoma is usually late, since the loss of vision often starts in the periphery and progression to the loss of central vision is late. Due to this, glaucoma is also called a silent thief of sight, with devastating consequences to the patient’s quality of life. Glaucoma is classified into two main types: primary and secondary glaucoma. Among primary glaucoma subtypes, primary open angle glaucoma (POAG) represents the major type of glaucoma affecting about 35 million people worldwide and is characterized by a juvenile or adult onset. Patients with POAG have characteristic glaucomatous optic nerve damage with corresponding visual field defects and an open anterior chamber angle at gonioscopy, but no other (congenital) anomalies [2, 3]. One of the significant risk factors for POAG is elevation of intraocular pressure (IOP). However, POAG also occurs in patients without elevated IOP, and an elevated IOP does not necessarily lead to POAG [4]. The gradual loss of the retinal ganglion cells (RGCs) is a hallmark of the disease along with the increased IOP, but the exact pathophysiological mechanisms of the disease are not fully understood. Well-studied risk factors associated with POAG include age, family history, gender, ethnicity, central corneal thickness, and myopia. In addition, genetic factors play an important role in the disease etiology.

To date, more than 15 loci have been identified for glaucoma, and the causative gene has been identified for 5 of these loci: *GLC1A* (*MYOC*/*TIGR)* [5, 6], *GLC1E* (*OPTN*) [7, 8], *GLC1F* (*ASB10*) [9, 10], *GLC1G* (*WDR36*) [11], and GLC1H (EFEMP1) [12, 13]. In addition, mutations in the *CYP1B1* [14] gene were identified in primary congenital, juvenile onset and adult onset POAG [15–17]. Finding the genes that cause glaucoma is the first step in improving early diagnosis and treatment of patients suffering from glaucoma. However, only less than 10% of POAG cases have pathogenic mutations in these disease-causing genes. It is therefore likely that the hereditary aspect of many of the remaining cases of POAG is either in the unidentified genes or due to the combined effects of several single nucleotide polymorphisms (SNPs). In recent years, several genome-wide association studies (GWAS) have identified several SNPs at different loci including *CAV1/CAV2* [18], *TMCO1* [19], *CDKN2B-AS1* [20], *CDC7-TGFBR3* [21], *SIX1/SIX6* [22], *GAS7*, *ATOH7*, *TXNRD2*, *ATXN2*, and *FOXC1* [23], to be associated with POAG, but they explain only a fraction of the disease heritability. In addition, the mechanisms how the associated loci influence the development of disease are often unclear. Therefore, additional genetic studies are required to explain the heritability, to gain a better understanding in the disease etiology, and to define new targets for treatment.

The goal of the current study was to identify the genetic cause of POAG in a large multiplex family using whole exome sequencing (WES).

## Materials and Methods

### Clinical Evaluation

A large family with eight individuals affected by POAG and one unaffected individual was ascertained. Affected and unaffected individuals were examined by an ophthalmologist at the Radboud University Medical Center in Nijmegen, the Netherlands. The study adhered to the principles of the Declaration of Helsinki and was approved by the Institutional Ethical Review Board of the Radboud University Medical Center in Nijmegen, the Netherlands. Blood samples were drawn from the family members, after obtaining written informed consent. DNA was extracted using standard methods.

Clinical characterization of the affected individuals included slit-lamp examination for iris diaphany, funduscopy, and IOP measurement with Goldmann applanation tonometry. Assessment of visual field defects was performed with a Humphrey Visual Field Analyzer (Carl Zeiss Humphrey Systems, Dublin, CA, USA). The decisions about glaucomatous damage on visual fields were based on the diagnostic criteria of the Hodapp et al. classification [24]. Evaluation of the anterior chamber angle was performed by gonioscopy, and corneal thickness was calculated by ultrasound pachymetry. In addition, a morphometric analysis of the optic disk was carried out by the Heidelberg Retina Tomograph II (HRT II; Heidelberg Engineering, Heidelberg, Germany), as described elsewhere [25]. An ophthalmic photographer masked to the results of the previous tests conducted the examination. The HRT Moorfields regression analysis (MRA) was used for classification of the optic disk [26]. The diagnosis of POAG was made when the following criteria were met: IOP higher than 22 mmHg (as measured by applanation tonometry in both eyes), glaucomatous optic neuropathy present in both eyes at funduscopy, visual field loss consistent with assessed optic neuropathy in at least one eye, and an open anterior chamber angle by gonioscopy.

### Whole Exome Sequencing and Analysis

**Fig. 1:**
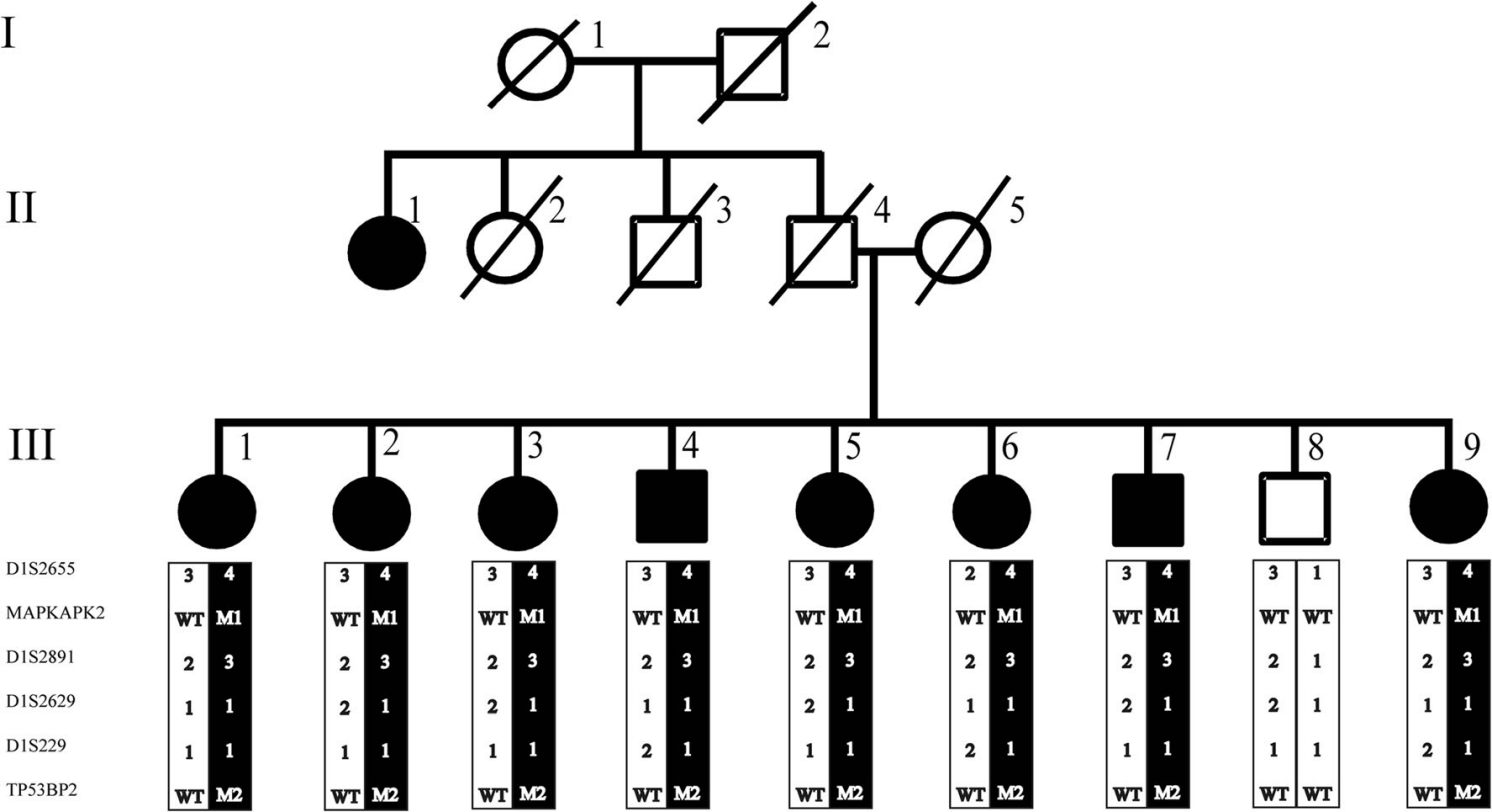
Pedigree of a family with individuals affected by POAG. The (c.109G>A; p.Val37Met) variant in the *TP53BP2* gene is indicated with M2, the variant (c.305G >A; p.Arg102His) in the *MAPKAPK2* gene is indicated with M1, and the wild type allele is indicated with WT for both genes together with microsatellite markers haplotype. All affected individuals carry both variants heterozygously, while the unaffected individuals do not carry the variant

To identify the underlying genetic cause of the disease in this large family with POAG, WES was performed using genomic DNA of four affected individuals (III:1, III:5, III:6, and III:8) (Fig. 1). Enrichment of exonic sequences was achieved by using the SureSelectXT Human All Exon V.2 Kit (50 Mb) (Agilent Technologies, Inc., Santa Clara, CA, USA). Sequencing was performed on a SOLiD 4 sequencing platform (Life Technologies, Carlsbad, CA, USA). The hg19 reference genome was aligned with the reads obtained using SOLiD LifeScope software V.2.1 (Life Technologies).

To identify the causative variant, only the variants shared by the four affected individuals were included for further analysis. All variants present within intergenic, intronic, and untranslated regions and synonymous substitutions were excluded. Variants present in the public genetic variant databases, including the Exome Variant Server (http://evs.gs.washington.edu/EVS/), dbSNP132 (http://www.ncbi.nlm.nih.gov/projects/SNP/snp_summary.cgi?build_id=132), and 1000 Genomes (http://www.1000genomes.org/) with an allele frequency >0.5%, were excluded.

To evaluate the pathogenicity of the variants obtained from WES, bioinformatic analysis was performed using the PhyloP (nucleotide conservation in various species) and Grantham scores (difference in physicochemical nature of amino acid substitutions). Functional predictions were performed using publically available tools, i.e., SIFT (http://sift.bii.a-star.edu.sg/ Sorting Intolerant from Tolerant), MutationTaster (http://www.mutationtaster.org/), and PolyPhen-2 (http://genetics.bwh.harvard.edu/pph2/ Polymorphism Phenotyping). Confirmation of variants and segregation analysis in all available family members was performed using PCR and Sanger sequencing. Sequencing was performed using the Big Dye Terminator Cycle Sequencing-Ready Reaction Kit (Applied Biosystems) on a 3730 DNA automated sequencer (Applied Biosystems, Foster City, CA, USA) using standard protocols. Segregating variants were analyzed in 180 population-matched controls by restriction fragment length analysis.

### Linkage Analysis

Microsatellite markers with a genetic heterogeneity >60% were selected from the UCSC database. Microsatellite markers D1S2655, D1S2891, D1S2629, and D1S229 were amplified with M13 tailed primers, followed by a second PCR with fluorescently labeled M13 primers. Fluorescent amplification products were visualized on the ABI-310 genetic analyzer, and the size of the alleles was determined with 500LIZ (Applied Biosystems, Bleiswijk, the Netherlands) and analyzed with the GeneMapper software version 3.7 (Applied Biosystems, Bleiswijk, the Netherlands). Multipoint linkage analysis was performed for informative markers to determine the logarithm of the odds (LOD) score using the GeneHunter program (version 2.1).11 in the easyLINKAGE Software package (http://nephrologie.uniklinikum-leipzig.de/nephrologie.site,postext,easylinkage,a_id,797.html). For linkage analysis, autosomal dominant inheritance was assumed, with a disease-allele frequency of 0.0001 and 95% penetrance.

## Results

### Clinical Evaluation

**Fig. 2:**
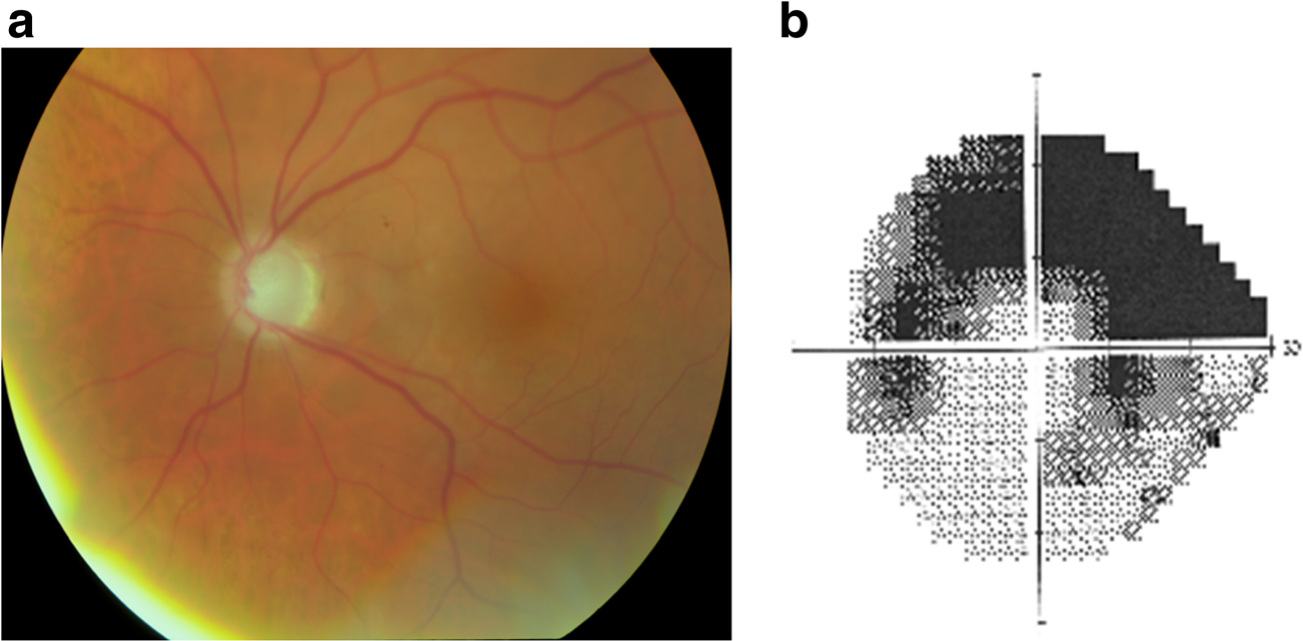
Photograph of the optic disk (**a**) and Humphrey visual field testing (**b**) of the left eye in a 76-years-old patient (III-9) with POAG and a corresponding visual acuity of 20/32. **a** Photograph shows a pallor, glaucomatous excavated optic disk. **b** Visual field testing shows a superior arcuate scotoma as well as inferior defects that are congruent with the excavation of the optic disk

Table 1 provides detailed clinical information of the eight affected family members diagnosed with POAG. All individuals had open drainage angles on gonioscopy (at least Shaffer grade III) and normal results of corneal thickness evaluation. The mean age at diagnosis was 54.8 years (range 49–60 years), with a mean IOP of 13.2 ± 2.2 mmHg (after use of IOP lowering medications). All individuals had bilateral glaucoma: they had glaucomatous optic neuropathy on funduscopy with reproducible compatible glaucomatous visual field loss, and all individuals showed abnormal results on Heidelberg Retina Tomograph II testing. A representative color photo of the optic disk of individual III-9 with POAG, with the corresponding superior arcuate scotoma on Humphrey visual field testing is shown in Fig. 2. From the medical chart, we distilled that the mean highest IOP recorded on diurnal testing was 23.6 ± 4.7 mmHg.

**Table 1:** Clinical features of patients with primary open angle glaucoma with the *TP53BP2* mutation

Patient number	Gender	Age at participation	Age at diagnosis	Presenting IOP	Laterality	Iris diaphany	Filtering surgery	Visual field loss at HFA^a^	MRA grade at HRT^b^	Presenting MD (dB) on Humphrey perimetry*	Presenting CPSD (dB) on Humphrey perimetry*
III:1	Female	78	49	13	Bilateral	No	Yes	In both eyes	Outside normal limits ODS	−30.49	4.05
III:2	Female	66	53	15	Bilateral	Yes	Yes	In both eyes	Borderline OD/outside normal limits OS	−24.58	11.00
III:3	Female	74	?	16	Bilateral suspect	No	No	OD	Outside normal limits OD/borderline OS	−3.90	3.05
III:4	Male	71	54	11	Bilateral	No	Yes	In both eyes	Outside normal limits ODS	−30.69	4.00
III:5	Female	70	54	9	Bilateral	Yes	Yes	In both eyes	Outside normal limits ODS	−31.10	3.11
III:6	Female	68	50	14	Bilateral	No	No	In both eyes	Borderline OD/outside normal limits OS	−15.82	12.87
III:7	Male	76	61	15	Bilateral	No	Yes	In both eyes	Outside normal limits ODS	−17.44	11.52
III:9	Female	79	63	13	Bilateral	No	No	In both eyes	Outside normal limits ODS	−9.99	8.02
Mean ± SD		72.7 ± 4.4	54.8 ± 4.8	13.2 ± 2.2							

IOP as measured by applanation tonometry in both eyes, presence of typical glaucomatous optic neuropathy with compatible visual field loss, open drainage angels on gonioscopy, and the absence of a secondary cause for glaucomatous optic neuropathy

*IOP* intraocular pressure, *Presenting IOP* highest IOP of two measurements in both eyes, *HFA* Humphrey Field Analyzer, *MRA* Moorfields regression analysis, *HRT* Heidelberg Retina Tomograph II, *SD* standard deviation, *OD* right eye, *OS* left eye, *ODS* both eyes

*Of worst affected visual field

^a^Visual filed loss on HFA, as defined by the HODAPP classification [24]

^b^MRA has three grades: “within normal limits,” “borderline,” and “outside normal limits”

### Mutation Detection

**Fig. 3:**
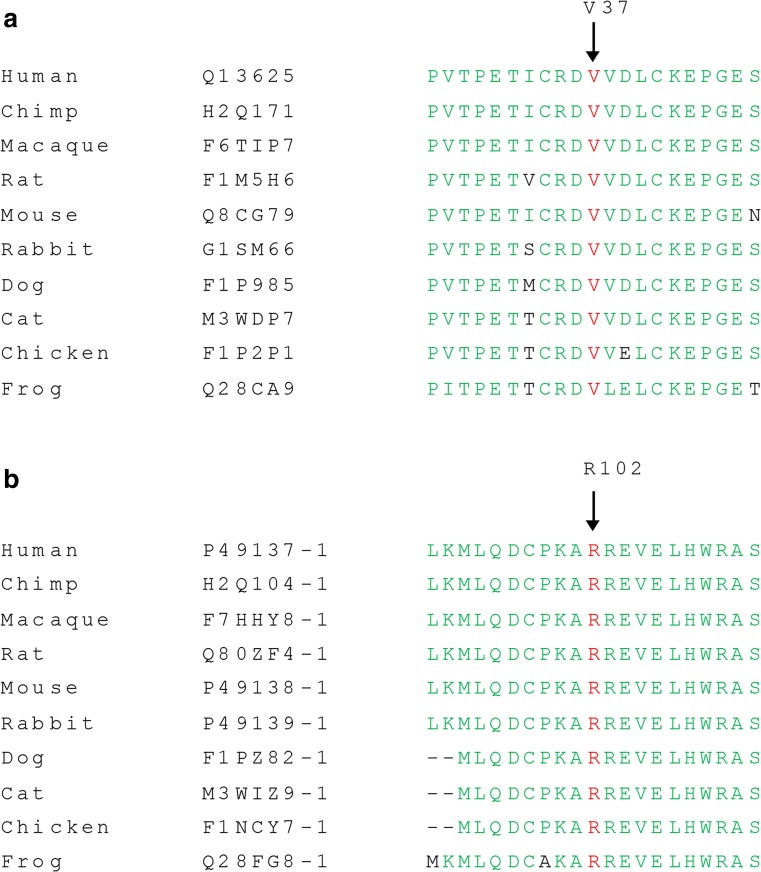
**a** Evolutionary conservation of valine at position 37 is represented by alignment of the human TP53BP2 (ASPP2) protein sequence to orthologous protein sequences of various vertebrate species. **b** Evolutionary conservation of arginine at position 102 is represented by alignment of the human MAPKAPK2 protein sequence to orthologous protein sequences of various vertebrate species

In Table 2, the number of variants that passed the various filtering steps per individual for the four affected individuals, as well as the variants shared by all four affected individuals is shown. The mean coverage of the WES data was 100X. Filtering for variants shared between all four affected individuals (III:1, III:5, III:6, and III:8) resulted in nine variants for further analysis (Table 3). Segregation analysis was performed for nine variants with a phyloP >2.7 or a Grantham score >80 (Table 3). Two novel heterozygous variants in the *TP53BP2* and *MAPKAPK2* genes were found to be segregating with the disease in the family (Fig. 1). The variant in the *TP53BP2* gene (c.109G>A; p.Val37Met) was predicted to be deleterious by SIFT, probably damaging by PolyPhen-2 and disease-causing by Mutation Taster (Table 3). The wild type nucleotide was highly conserved (phyloP score 4.08), and the amino acid residue p.Val37 was completely conserved among vertebrates (Fig. 3a). The second variant that segregated with the disease in the family was identified in the *MAPKAPK2* gene (c.305G>A; p.Arg102His) (Fig. 1). This variant was also predicted to be deleterious by SIFT, probably damaging by PolyPhen and disease-causing by Mutation Taster (Table 3). The wild type nucleotide was highly conserved (phyloP score 5.69), and the amino acid residue p.Arg102 is completely conserved among vertebrates (Fig. 3b). Both variants were not identified in 180 population-matched controls and were not present in the Exome Variant Server, dbSNP132 and 1000 genomes.

**Table 2:** Number of variants identified per individual and shared between four affected individuals

Filtration steps	Individual 1	Individual 2	Individual 3	Individual 4	Variants shared by all 4 individuals
Total number of variants	45.755	47.697	45.417	41.943	21.447
SNP frequency <0.5	28.789	30.381	28.614	26.252	9.065
In-house database frequency <0.5	2.152	2.529	2.503	2.373	55
Exonic and canonical splice site variants	796	873	961	987	33
Nonsynonmous variants	535	620	682	696	18
Grantham score >80	248	268	302	323	4
Phylop >2.7	22	31	24	22	9

**Table 3:** Rare variants shared by four affected individuals and segregation analysis

Gene ID	Protein isoform	cDNA position	Amino acid position	phyloP	Segregation	Grantham score	SIFT	Mutation Taster	PolyPhen-2
*TP53BP2*	NM_001031685	109C>T	p.Val37Met	4.135	Yes	21	Deleterious	Disease causing	Probably damaging
*MAPKAPK2*	NM_032960	305G>A	p.Arg102His	5.691	Yes	29	Deleterious	Disease causing	Probably damaging
*TGFBI*	NM_000358	895G>A	p.Asp299Asn	5.884	No	23	Tolerated	Disease causing	Probably damaging
*ADSSL1*	NM_199165	578C>T	p.Ser193Phe	5.657	No	155	Deleterious	Disease causing	Probably damaging
*NCEH1*	NM_001146276	142C>T	p.Ala48Thr	5.305	No	58	Tolerated	Disease causing	Probably damaging
*RNF157*	NM_052916	1913C>G	p.Cys638Ser	5.254	No	112	Tolerated	Disease causing	Probably damaging
*PHC3*	NM_024947	1840C>T	p.Glu614Lys	3.961	No	56	Tolerated	Disease causing	Possibly damaging
*SLITRK3*	NM_014926	2581G>A	p.Arg861Cys	3.886	No	180	Deleterious	Disease causing	Possibly damaging
*RYR2*	NM_001035	8162 T>C	p.Ile2721Thr	3.683	No	89	Deleterious	Disease causing	Possibly damaging

### Linkage Analysis

Twelve microsatellite markers were used for linkage analysis of the genomic region encompassing the *MAPKAPK2* and *TP53BP2* genes, but only four markers were informative. A multipoint LOD score of 2.48 was obtained for markers D1S2655 and D1S2891, which is suggestive of linkage and is in accordance with the maximum LOD score that can be achieved considering the structure of the pedigree. The disease-associated haplotype encompasses markers D1S2655, the *MAPKAPK2* variant, D1S2891, D1S2629, D1S229, and the *TP53BP2* variant. All affected individuals carry the disease haplotype that includes both genetic variants, which indicates that both variants are in the same linkage interval and are in *cis* configuration.

## Discussion

In the current study, we used WES to identify the genetic defect in a large family with POAG. We identified potentially pathogenic variants in the *TP53BP2* (c.109G>A; p.Val37Met) and *MAPKAPK2* (c.305G>A; p.Arg102His) genes. A disease haplotype carrying both variants segregates with the disease, with a maximum LOD score of 2.4. Variants in both *TP53BP2* and *MAPKAPK2* segregate with the disease, since both of them are on the same haplotype. Therefore, segregation of the *MAPKAPK2* variant with the disease is a coincidental finding. Since the inheritance pattern was not obvious from the pedigree, we considered both recessive and dominant inheritance patterns during the variant prioritization process. However, no homozygous or compound heterozygous variants were identified among the putative pathogenic variants that were shared between affected individuals, and this thus does not support recessive inheritance. Since phenotype data and DNA were not available from the deceased parents, we can only speculate that the inheritance pattern may be autosomal dominant.

*TP53BP2* encodes a member of the ASPP (apoptosis-stimulating protein of p53) family of p53-interacting proteins comprised of three members: ASPP1, ASPP2, and iASPP. Both ASPP1 and ASPP2 are proapoptotic proteins involved in the regulation of the apoptosis and are encoded by the *TP53BP2*, and iASPP is encoded by *PPP1R13B* genes, respectively [27]. ASPP2 is well known for its binding and activation of the apoptotic function of p53, p63, and p73 by selectively enhancing their DNA-binding and transactivation activities on proapoptotic genes such as *BAX* and *PIG3* [27, 28]. Apoptosis is tightly regulated during normal development. In the case of abnormal regulation, it mediates cell death of neuronal cells in neurodegenerative diseases such as Alzheimer’s disease or Parkinson’s disease or death of RGCs in glaucoma due to overexpression of p53 [29–31]. Recently, the role of ASPP1 and ASPP2 proteins in neuronal apoptosis and their involvement in the regulation of adult RGCs after injury have been investigated. The results indicated that both ASPP1 and ASPP2 are highly expressed in RGCs and contribute to p53-dependent death of RGCs.

In glaucoma cell death of the post-mitotic neurons, i.e., RGCs, occurs due to an increased rate of apoptosis. The ASPP proteins are involved in the regulation of apoptosis by activating p53. The expression of ASPP2 affects the DNA binding activity of p53 on the Bax promoter or downstream targets involved in apoptosis [32]. In the current study, we speculate that binding between ASPP2 and p53 may be affected by an amino acid variant (c.109G>A; p.Val37Met) in the ASPP2 protein, which leads to the increased accumulation of p53, followed by an increase in cell death of the RGCs, subsequently leading to glaucoma. Previously, it has been reported that normal ASSP2 protein is required for the activation of apoptosis in a controlled manner. It was observed that the blockade of the ASPP-p53 pathway is important for the survival of neurons after axonal injury [33]. The results of Wilson et al. are further supported by a recent study in an in vivo model of acute optic nerve damage, in which it was shown that iASPP is expressed by injured RGCs and short interference RNA (siRNA)-induced iASPP knockdown exacerbates RGC death, while RGC survival was enhanced by adeno-associated virus (AAV)-mediated iASPP expression. Increased expression of iASPP in RGCs downregulates p53 activity and blocks the expression of proapoptotic targets PUMA and Fas/CD95 [34]. Since iASPP is an inhibitor of p53-mediated apoptosis, it is possible that the mutation in the ASPP2 protein influences the expression of iASPP. Subsequently, it would not be able to perform actively in the survival of retinal ganglion cells due to apoptosis.

In a recent study, it has been observed that siRNA interfering the expression of ASPP2 is involved in the development of the proliferative vitreoretinopathy (PVR). Using epiretinal membranes of PVR patients, they examined the expression of ASPP2 using immunohistochemistry and observed reduced expression of ASPP2 in PVR membranes. In addition, knockdown of ASPP2 is involved in increased expression of cytokines such as TGF-*β*, CTGF, VEGF, TNF-*α*, and interleukins [35]. In glaucoma, the role of inflammatory cytokines is well known, and it is possible that the amino acid variant identified in the ASPP2 protein affects the expression of inflammatory cytokines and interleukins, which mediate apoptosis of retinal ganglion cells in glaucoma. In another recent study, the neuroprotective effect of minocycline in rats with glaucoma was evaluated, and downregulation of *TP53BP2* was observed upon treatment [36]. Minocycline is a tetracycline with anti-inflammatory and anti-apoptotic properties. In previous studies, it has been shown that minocycline significantly delays RGC death in models of experimental glaucoma and optic nerve transaction [37].

Taken together, these studies support the involvement of the *TP53BP2* gene in glaucoma and suggest that the genetic variant identified by WES in the large POAG family may be relevant to the disease.

The second variant that segregates with the disease in the family was identified in MAPK-activated protein kinase 2 (MAPKAPK2, also known as MK2), which is one of the downstream targets of p38 MAPK. The Ocular Tissue Database (OTDB, http://genome.uiowa.edu/otdb/) demonstrates a minimal expression in the eye for MAPKAPK2 in contrast to TP53BP2. Therefore, *TP53BP2* gene seems to be the strongest candidate to be associated with the disease in this particular family.

In conclusion, through WES in a large POAG family, we identified a novel genetic variant in the *TP53BP2* gene, which is predicted to be pathogenic and affects a highly conserved amino acid residue. Since it has been demonstrated that the gene regulates apoptosis in RGCs and is downregulated upon minocycline treatment in a glaucoma rat model, *TP53BP2* may represent a novel gene associated with POAG. Additional screening of the *TP53BP2* gene in other familial and sporadic patients with POAG from different populations is required to confirm its involvement in the disease.
